# Redundancy-free integrated optical convolver for optical neural networks based on arrayed waveguide grating

**DOI:** 10.1515/nanoph-2023-0513

**Published:** 2024-01-02

**Authors:** Shiji Zhang, Haojun Zhou, Bo Wu, Xueyi Jiang, Dingshan Gao, Jing Xu, Jianji Dong

**Affiliations:** Wuhan National Laboratory for Optoelectronics, Huazhong University of Science and Technology, Wuhan 430074, China; Optics Valley Laboratory, Wuhan 430074, China

**Keywords:** optical computation, optical neural network, convolution, silicon photonics

## Abstract

Optical neural networks (ONNs) have gained significant attention due to their potential for high-speed and energy-efficient computation in artificial intelligence. The implementation of optical convolutions plays a vital role in ONNs, as they are fundamental operations within neural network architectures. However, state-of-the-art convolution architectures often suffer from redundant inputs, leading to substantial resource waste. Here, we demonstrate an integrated optical convolution architecture that leverages the inherent routing principles of arrayed waveguide grating (AWG) to execute the sliding of convolution kernel and summation of results. *M* × *N* multiply–accumulate (MAC) operations are facilitated by *M* + *N* units within a single clock cycle, thus eliminating the redundancy. In the experiment, we achieved 5 bit precision and 91.9 % accuracy in the handwritten digit recognition task confirming the reliability of our approach. Its redundancy-free architecture, low power consumption, high compute density (8.53 teraOP mm^−1^ s^−1^) and scalability make it a valuable contribution to the field of optical neural networks, thereby paving the way for future advancements in high-performance computing and artificial intelligence applications.

## 1 Introduction

Convolutional neural networks (CNNs) have become indispensable in various applications, such as image recognition and natural language processing [1–3]. The adoption of CNNs has grown exponentially, creating higher demands for efficient hardware implementations to maximize throughput and overall efficiency. Importantly, convolutional layers account for over 90 % of computations in most CNN architectures [4].

However, the hardware deployment efficiency of convolutions faces significant challenges due to inherent redundancy and computational waste in convolution calculations [5]. The redundancy emerges from the convolution operation’s overlapping nature, involving numerous multiplications and accumulations on identical input samples. This occurs when a small kernel is sliding across a large dataset, such as an image. At each position, a series of multiplications is carried out, and their results are summed. The issue arises due to the kernel’s overlap with neighboring data segments, leading to repeated multiplications and accumulations for these overlapping areas. Using multiple devices or clock cycles for these calculations yields ineffective resource utilization, restraining real-time processing capabilities. Addressing these issues becomes crucial as computational demands continue to surge, presenting formidable challenges to current computational hardware platforms.

In response to the limitations of electronic computation, optical neural networks have emerged as one of the most competitive candidates in the next-generation computing hardware platform. Photonic chips leverage the ultra-wide bandwidth of optoelectronic devices, enabling them to achieve clock frequencies 1–2 orders of magnitude higher than existing electronic chips [6, 7]. The various physical dimensions of light, including wavelength, mode, and polarization, offer substantial computational parallelism, leading to a manifold improvement in computational efficiency [8, 9]. An essential advantage of optical computation lies in its propagation-as-computation of light [10, 11], which allows for ultra-low latency far beyond the capabilities of traditional electronic chips [12, 13]. This unique attribute opens up exciting possibilities for novel applications, such as autonomous driving and ultrafast science [14, 15].

Despite several demonstrations of optical convolutions attempting to utilize the advantages of optical computing mentioned above, they may have some degree of limitations that need to be addressed. Solutions based on spatial light modulator [16–18] encounter limitations related to the bulky size of discrete devices, which hampers effective integration. Metamaterial and on-chip diffraction approaches [19, 20] have practicality issues due to the non-reconfigurability of the convolution kernel. Dot product schemes [13, 21] suffer from redundancy in convolution manipulation, necessitating numerous redundant devices and resulting in extremely low computational efficiency. Delay line-based schemes [22–25] face challenges in system scalability as they require a large number of huge-sized delay lines, and the convolution operation takes multiple clock cycles to execute due to the delay. Although on-chip spatial Fourier transform scheme [26] reduces the device size and number effectively, they can only handle cyclic convolution for input and kernel sizes that are the same, which significantly deviates from the application scenarios of convolutional neural networks. Synthetic dimension scheme [27] demands high-speed modulation to achieve static convolution kernels, leading to a substantial increase in power consumption. Importantly, most of the mentioned schemes are to some extent limited by the inherent redundancy in convolution computation, resulting in a decrease in computation efficiency.

Here, we present a redundancy-free on-chip optical convolution scheme based on arrayed waveguide grating (AWG). It entails encoding input information into intensities at various wavelengths and broadband intensity modulators are positioned at different input ports to represent the convolution kernel. Subsequently, wavelength routing occurs through an AWG chip fabricated on the silicon-on-insulator (SOI) platform. Finally, the convolution results are obtained at the output ports. This approach effectively overcomes the challenges mentioned earlier, enabling convolution to be implemented without redundancy. For an input vector of size *N* and a kernel size of *M*, our scheme requires only *N* modulators for input vector encoding and *M* broadband modulators for kernel encoding, accomplishing computation in one clock cycle. The hardware utilization efficiency has theoretically reached its maximum. In our design, energy consumption occurs solely during the encoding of the input vector and convolution kernel, where we have minimized the number of devices. The convolution process itself remains entirely passive with nearly-zero power consumption. Notably, since intensity summation takes place at the output ports, coherence is unnecessary. This not only reduces the overall system complexity and costs but also offers potential support for cascading multiple layers of optical neural networks. The reliability of our scheme is validated through experimental verification, achieving 5 bit precision and 91.9 % accuracy rate on a handwritten digit classification task using the Modified National Institute of Standards and Technology (MNIST) dataset. Additionally, we discuss the comparative advantages of our approach compared to other on-chip reconfigurable solutions. The theoretical computing power density of our scheme can reach 8.53 TOPS mm^−2^, which is comparable to the state-of-the-art metrics [28]. This significant advancement marks a stride forward in the realm of integrated all-optical neural networks, leading to the emergence of next-generation computing platforms.

## 2 Principle and device design

The mathematical form of convolution is illustrated as follows: Consider an input signal *x*(*n*) of length *N*, where *n* represents the discrete index ranging from 0 to *N* − 1. Similarly, we have a kernel *h*(*m*) of length *M*, where m represents the discrete index ranging from 0 to *M* − 1. The convolution operation can be expressed as:

(1) $$y\left(n\right)=\overset{M-1}{\underset{m=0}{\sum }}x\left(n-m\right)\times h\left(m\right)$$

Here, *y*(*n*) represents the output signal at index *n*, which is obtained by multiplying each element of the input signal *x*(*n*) with the corresponding element of the kernel *h*(*m*), and then summing up the results. The index *m* represents the shifting or sliding of the kernel over the input signal for convolution. The output signal *y*(*n*) will have a length of *N* + *M* − 1.

**Figure 1:**
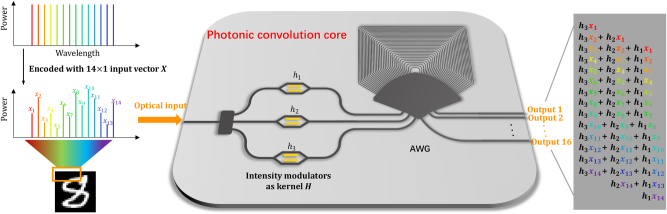
Operation principle of the photonic convolution core with AWG chip.

Figure 1 shows the photonic convolution core, which loads input vector in frequency (or wavelength) domain, loads kernel in modulator array and distributes convolution results to AWG output ports in spatial domain. The input data, represented by a vector *X* of length *N*, is encoded into the intensity of various wavelengths from a multi-wavelength light source. The frequency interval, denoted as Δ*f*, matches the frequency spacing of the AWG. The convolution kernel, a vector *H* of length *M*, is encoded using *M* intensity modulators. This process generates *M* weighted multi-wavelength information, which is then input through *M* adjacent ports of the AWG and routed within it. Since the frequency spacing of the AWG matches that of the input vector, multiple wavelength information from the same input port propagates to different output ports. The sliding of the convolution kernel window is achieved through loading information from adjacent input ports and the unique routing property of the AWG. Information with different wavelengths, each carrying distinct convolution kernel weights, is routed through the AWG and combined at the output ports, resulting in the convolution output at *M* + *N* − 1 ports. The total number of MAC operations is *M* × *N*, with the number of operations given by 2 × *M* × *N*. Figure 1 illustrates the operational principle of the photonic convolution core with *N* = 14 and *M* = 3. Given the constraints of on-chip vertical optical fields, only a single spatial dimension remains after eliminating the direction of light propagation. This makes direct on-chip implementation of two-dimensional (2D) convolution a significant challenge. The principle of our architecture and the demonstration of our experiment are based on one-dimensional (1D) convolution. Nonetheless, a 2D convolution can be equivalently portrayed as a 1D convolution by applying zero-padding to the kernel [27]. With our method that encodes the convolution kernel using a wideband modulator at the input ports, the zero-padding merely involves omitting specific input ports. This negates the need to increase the count of modulated devices and ensures no redundancy is introduced during the 2D to 1D transition. Our presented approach is tailored to implement positive convolution kernels. To accommodate convolution kernels spanning real-number parameters, one can decompose a kernel into two separate entities, one with positive values and the other with negative. The final subtraction can be facilitated at the output ports using balanced photodetectors.

**Figure 2:**
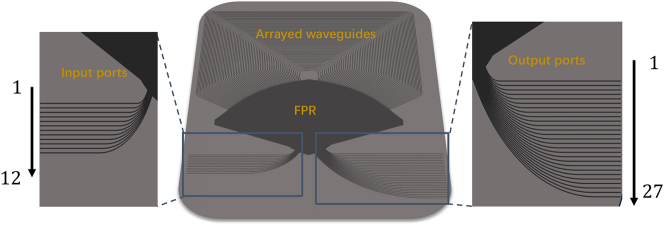
Schematic diagram of an overlapped star coupler AWG structure.

The AWG is based on a commercial SOI wafer with a 220 nm top silicon thickness and SiO_2_ top cladding. The AWG has a central wavelength of 1550 nm, a channel spacing of 100 GHz (i.e., 0.8 nm), and a free spectral range of 34.246 nm. Both the arrayed waveguides and free propagation region (FPR) are silicon ridge waveguides with an etch depth of 70 nm to reduce phase errors and sidewall scattering loss in the arrayed waveguides. The AWG structure is shown in Figure 2. It has 12 input channels and 27 output channels. The input and output star couplers are overlapped to make the device more compact, with a size of only 1.5 mm × 1.5 mm. There are 185 arrayed waveguides, each consisting of a bent waveguide and straight waveguide. The straight waveguide width is 1.2 μm, the bent waveguide width is 800 nm, and the bent radius is between 47 and 53 μm. Tapers are used between the straight and bent waveguides. The star coupler radius is 353.877 μm. The adjacent arrayed waveguide length difference is 19.394 μm, corresponding to a grating order *m* = 35. The waveguide spacing is 3 μm, both on the input/output star coupler circumference and on the AWG circumference. To reduce coupling loss between the arrayed waveguides and free propagation region, the arrayed waveguide width on the grating circumference is expanded to 2.8 μm. The input and output waveguides on the star coupler circumference have a width of 1.5 μm.

The input and output channel numbers of the AWG are shown in Figure 2. For a given input, lower numbered output ports output longer wavelengths, while higher numbered ports output shorter wavelengths. Based on the cyclic routing property of the AWG, for a fixed wavelength input, increasing the number of input port by one will cause the corresponding output port number for that wavelength to decrease by one.

The AWG features 12 input ports and has the capability to route 16 wavelengths, allowing it to support a maximum convolution size of *N* = 16 and *M* = 12. The AWG has 27 output channels to accommodate convolution size of *M* + *N* − 1. Note that the scale of the AWG can be further increased and Many studies have already demonstrated larger-scale AWGs [29–36]. As the number of input ports of the AWG is equivalent to the size of the convolutional kernel, and the number of wavelengths the AWG can demultiplex represents the size of the input vector. Previous work has presented the AWG with 512 input ports and 512 wavelengths [34], indicating that the size of the input vector and kernel could see an increase of one to two orders of magnitude beyond our current demonstration in the future. Here, our emphasis lies in exploring the untapped potential of AWG in optical computing. Despite not pushing the AWG’s scale to its limit in this work, the AWG chip we designed can still reach a remarkable compute density.

## 3 Results

**Figure 3:**
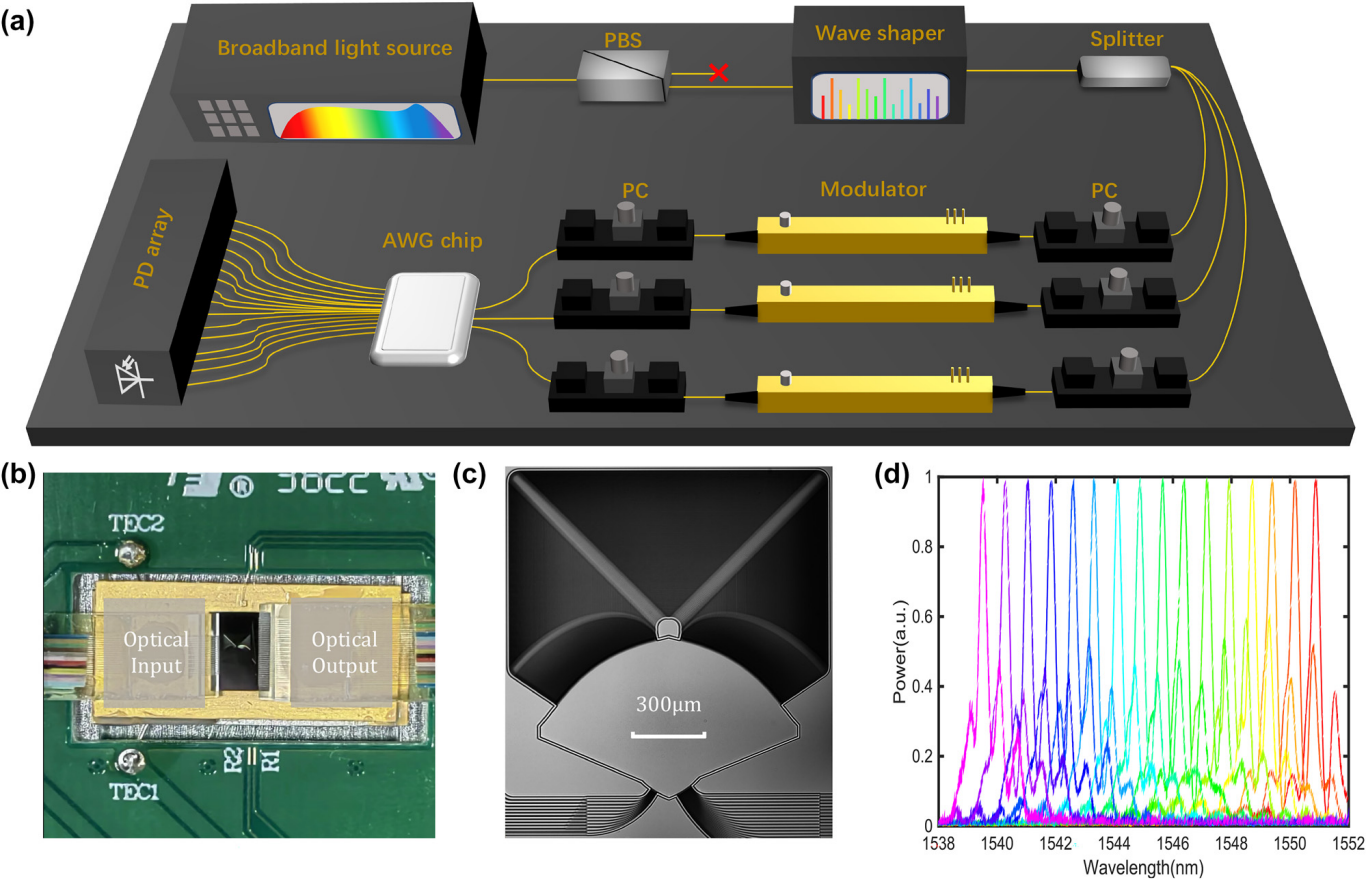
Experimental realization of the convolver. (a) Experimental setup. PC, Polarization controller; PBS, Polarizing beam splitter; PD, photodetector. (b) Overall photo of the packaged layout. The integrated photonic core is mounted on a thermoelectric cooler (TEC). The optical input and output (I/O) are through the fiber arrays on the left and right sides. (c) Microscopic image of the fabricated AWG. (d) Measured transmission spectra of 16 output ports for corresponding one input port.

Figure 3(a) shows the experimental setup. First, we make use of a broadband light source (Amonics ALS-CL-15) and an optical spectral shaper (Finisar WaveShaper 1000S) together to generate a multi-wavelength light source and encode the input vector. The selection of the passband frequency for the optical spectral shaper is determined based on the measured AWG chip spectrum shown in Figure 3(d). The spectrum is pre-measured using an optical spectrum analyzer (YOKOGAWA AQ6370C). Following that, the light power is divided into three equal parts using a beam splitter, and these three channels of signals are then fed into three intensity modulators (JDS Uniphase 21049397, the half-wave voltage is 5.9 V) to represent a 3 × 1 convolution kernel. After routing through the AWG chip, the light is measured using a photodetector array (LUSTER OPM-1008, InGaAs photodetector, the working wavelength is 1530–1570 nm and the sensitivity is −50 dBm) at the 16 output ports. The light polarization is controlled by a PBS and PCs before coupling into the intensity modulators and the grating coupler of the chip. The whole system is controlled by a custom programmable voltage source. Figure 3(b) shows the overall photo of the packaged layout, in which both thermoelectric cooler (TEC) and vertical grating coupling have already been packaged for temperature control and optical input/output (I/O). The microscopic image of the fabricated AWG is shown in Figure 3(c).

Even though the AWG chip can support a maximum of 16 wavelengths and 12 input ports, our experiment is constrained by the available equipment. Therefore, we employ an AWG to carry out the convolution with an input vector length of *N* = 14 and a convolution kernel size of *M* = 3. We select 14 channels out of the 16 available wavelengths shown in Figure 3(d), and select input Port 1 to Port 3 of AWG chip, as depicted in Figure 2. The results are then measured at output ports from No. 10 to No. 25.

In this section, we first characterized its computational precision and verified the reliability of our photonic convolution core. Next, we conducted an experimental demonstration of the handwritten digit classification task using optical CNN.

### 3.1 Characterization of compute precision

**Figure 4:**
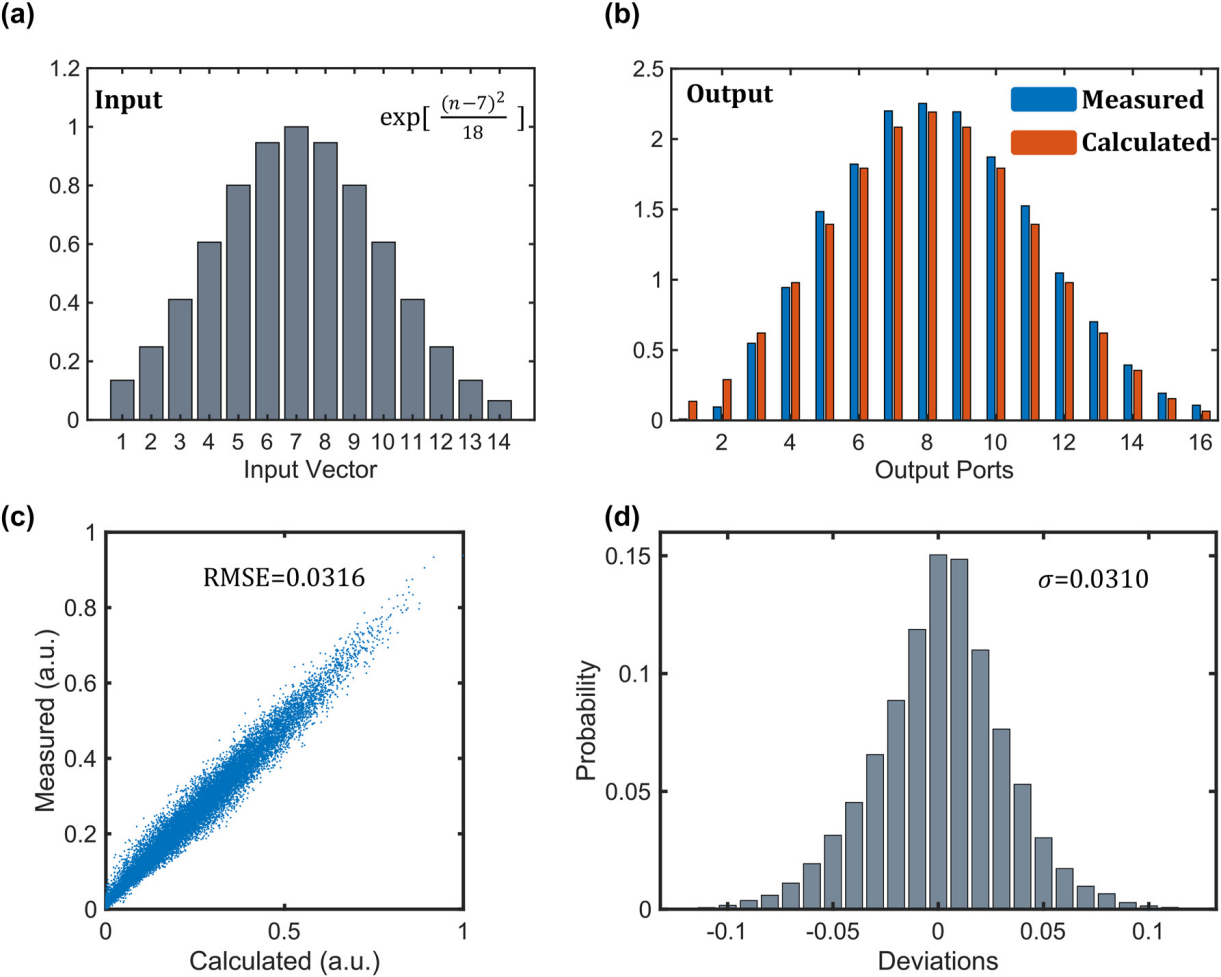
Precision characterization of the convolver. (a) A Gaussian distributed input vector. (b) The calculated and measured output results of the input vector after convolution with the kernel [1, 0.3, 1]. (c) Scatter plot for convolution accuracy measurement with 1000 random inputs and kernels, resulting in 16,000 data points. (d) Histogram of compute errors over 16,000 data samples.

In Figure 4(a) and (b), we employed a Gaussian distributed input vector and a [1, 0.3, 1] convolution kernel to visually demonstrate the effect of our convolution. Initially, we encoded the 14 wavelengths using the optical spectral shaper, resulting in a Gaussian distribution. Then, by applying voltage to the modulators, we modulated the transmittance of the three modulators to 1, 0.3, and 1, respectively, as per the convolution kernel. Finally, the measurement results were obtained from the detector array. The calculated and measured results are displayed in Figure 4(b).

We conducted tests on 1000 sets of random input vectors and convolution kernels, yielding 16,000 data points, as each convolution output had a length of 16. Each element of the input vector was a random number between 0 and 1, while each element of the convolution kernel was a random number between 0 and 1/3. This ensured that the output data points ranged from 0 to 1. For a total of *N* data points, the root mean square error (RMSE) was calculated as:

(2) $$\text{RMSE}=\sqrt{\frac{\overset{N}{\underset{i=1}{\sum }}{\left(p_{i}-q_{i}\right)}^{2}}{N}}$$

Here, *p*_*i*_ presents the *i*th data point of the measured data, and *q*_*i*_ represents the calculated result corresponding to the *i*th data point, resulting in an RMSE of 0.0316. Figure 4(d) illustrates the probability distribution of errors. As the bit precision *N*_*b*_ is expressed as follows:

(3) $$N_{b}=log_{2}\left(\frac{\mu_{\max}-\mu_{\min}}{\sigma }\right)$$

where *μ*_max_ and *μ*_max_ are the maximum and minimum values of the output, respectively. *σ* is the standard deviation of the errors between the experimental output and expected output. Since our output range is 0–1, with an error standard deviation of 0.0310, the computational precision of the system is determined to be 5 bits. Indeed, this result is comparable to the precision of the majority of photonic computing architectures. A 5 bit precision is already adequate for many neural network inference scenarios.

### 3.2 Demonstration of handwritten digits classification

**Figure 5:**
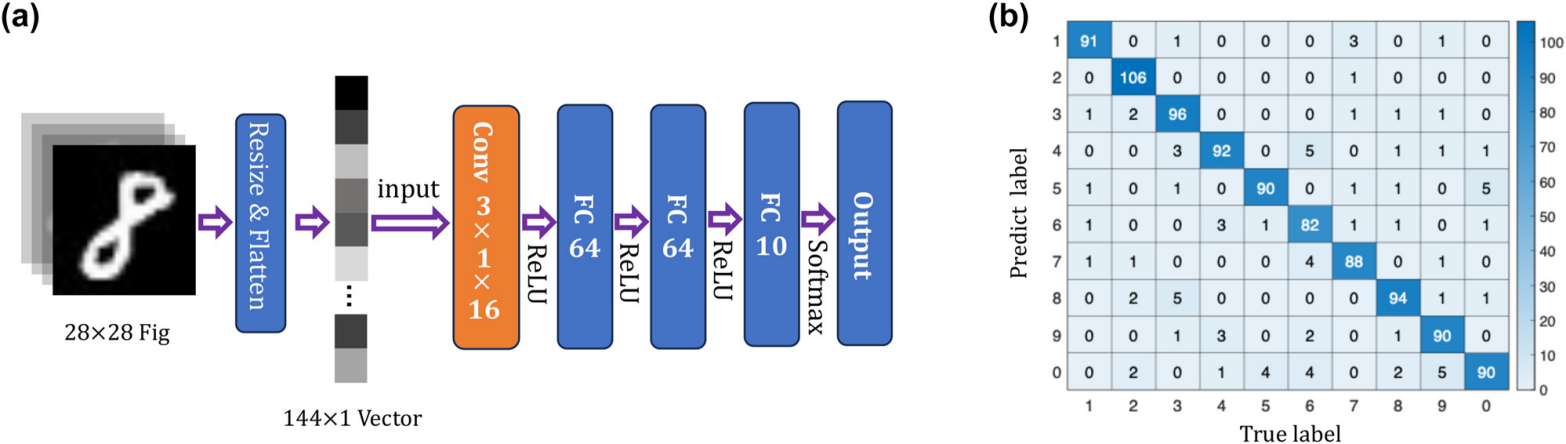
Experimental results of a CNN. (a) The network structure of the CNN, where the orange part represents the optically implemented convolutional layer. (b) The confusion matrix of recognizing 1000 digits in the MNIST test database.

In Figure 5(a), we employed a CNN for ten-class classification of “0–9” hand-written digit images, where the convolutional layer is implemented using photonic convolutional core. The images are first resized to 12 × 12 and then flattened into a 144 × 1 vector. The photonic convolutional layer utilizes sixteen 3 × 1 convolutional kernels, generating sixteen 146 × 1 feature maps. To achieve the convolution of the long vector, it is converted into multiple short vector convolutions, which are subsequently encoded for optical execution. After applying the ReLU non-linear activation function, the sixteen 146 × 1 feature maps are reshaped into a 2336 × 1 vector. Considering that our approach currently only supports positive convolution kernels, we impose a constraint during the training process, limiting the parameters of the convolution layers to be positive.

The vector then passes through two fully connected layers, each with 64 neurons activated by the ReLU function. The final layer consists of 10 neurons, activated by softmax, representing the output layer for classification. Offline training using the backpropagation algorithm (stochastic gradient descent) is performed to minimize cross-entropy loss. We conducted ten-class classification on 70,000 images from the MNIST dataset, using 60,000 images for training and a 5:1 split between the training and validation sets. Additionally, 10,000 images are used for testing. After 20 epochs, the theoretical accuracy reaches 95.1 %.

The experiment utilized the trained parameters to infer 1000 test images and achieved an accuracy of 91.9 %, as shown in the confusion matrix in Figure 5(b). The deviation of 3.2 % from the theoretical accuracy is primarily attributed to the limited bit precision. It’s worth noting that quantization was only applied during the final inference stage. Introducing quantization earlier, during the training process is expected to substantially reduce this deviation. The constraint in bit precision arises from various factors, including polarization state jitter, temperature drift, and other sources of noise. However, these issues can be mitigated through various feedback adjustments [37–40] and *in-situ* training [10, 41–44].

## 4 Discussion

Table 1 summarizes the comparison of our photonic convolution scheme with other mainstream reconfigurable on-chip schemes in terms of several metrics. We have chosen the performance in scenarios with input vector length *N* and convolution kernel size *M* as our comparative metric. In our approach, we treat the input vector as rapidly updated, while the convolution kernel is slowly refreshed. As a result, the input vector needs to be encoded using high-speed modulation devices, whereas the nearly static convolution kernel can be modulated using low-speed devices.

**Table 1:** Comparison among reconfigurable on-chip schemes.

	Delay lines [24, 25]	Synthetic dimension [27]	Fourier transform [26]	Dot product [13, 21]	AWG (this work)
Number of fast modulated devices	1	*N* + 1	*M* + *N* − 1	*M*	*N*
Number of slow modulated devices	*M*	0	*M* + *N* − 1	*M*	*M*
Total number of modulated devices	*M* + 1	*N* + 1	2 × (*N* + *M* − 1)	2 × *M*	*M* + *N*
Number of clock cycles	*N* + *M* − 1	N/A	1	*N* + *M* − 1	1
Product of modulated devices and clock cycles	(*M* + 1) × (*N* + *M* − 1)	N/A	2 × (*N* + *M* − 1)	2 × *M* × (*N* + *M* − 1)	*M* + *N*

Our comparative metrics include the number of high-speed modulation devices, the number of low-speed modulation devices, and the clock cycles required to complete the convolution operation. To represent the overall computing resource utilization, we multiply the total number of devices with the clock cycles, where a lower value indicates higher comprehensive computing efficiency for the proposed approach.

It is essential to note that the synthetic dimension convolution scheme is not suitable for this comparison. This is because its clock cycles should be defined based on the sampling rate of modulating the convolution kernel rather than the refresh rate of the input vector. The synthetic dimension convolution scheme requires a sampling rate much higher than the input vector update speed for modulation to obtain a static convolution kernel. Consequently, this results in significant energy waste, making it unsuitable for fair comparison with other approaches.

Compared with previous works, our scheme has several advantages:

(a) Our approach achieves the minimal product of device count and clock cycles, reducing this value to the theoretical minimum. As a result, our approach stands as the only on-chip convolution scheme without redundancy, making it unique and highly efficient.
(b) The entire convolution operation can be completed in just one clock cycle. By capitalizing on the benefits of ultra-low latency in optical computation, our approach effectively leverages the ability of light to perform computation during propagation. This characteristic creates substantial opportunities for applications that place a strong emphasis on low latency, providing distinctive advantages in such scenarios.
(c) Allowing the size of kernels to be smaller than inputs. For CNNs, the kernel size is typically much smaller than the size of input. Small convolution kernels are widely used due to their effectiveness in capturing local features [45], particularly in image processing applications. The Fourier transform scheme has a limitation in its applicability. It requires the input and convolution kernel sizes to be the same, significantly restricting its potential application scenarios.
(d) The ability to cascade with fully connected layers. Existing convolutional neural network architectures commonly involve cascading convolutional layers followed by fully connected layers. However, the delay-line-based approach outputs temporal sequence information, and optical fully connected layers for such temporal sequences are currently unavailable. Similarly, Fourier-transform-based approaches require coherence, which is prone to loss due to noise in cascaded multi-layer networks. In contrast, our approach generates spatial information without any coherence requirements. This characteristic allows it to be highly compatible with the majority of current optical nonlinear activation functions [13, 46–49] and optical fully connected layers [50, 51], facilitating seamless integration into all-optical neural network architectures.

The most distinctive aspect of our optical convolution core is that the number of multiply-add operations is *M* × *N*, while the number of implemented modulation devices is *M* + *N* (including high-speed and low-speed devices). As power consumption solely occurs in the modulation devices, the power consumption per operation sharply decreases as the scale of *M* and *N* increases, leading to a substantial increase in compute density as well.

Throughout this paper, our primary focus is to highlight the immense potential of AWG for optical convolution operations, and we did not intentionally pursue the scale of AWG. Nevertheless, our AWG chip can still theoretically achieve an astonishing compute density of 8.53 TOPS/mm^2^, and the calculation method is as follows:

(4) $$\begin{array}{c} \frac{2\times 12\times 16\times 50\;\;GHz}{1.5\;\;mm\times 1.5\;\;mm}=8.53\;\;TOPS\;\;mm^{-2} \end{array}$$

In this calculation, the number 16 represents the maximum number of wavelengths, which corresponds to the maximum input vector size. Similarly, the number 12 represents the maximum number of input ports, which is the size of the convolution kernel. The factor of two in the multiplication accounts for the fact that one multiply-add operation involves two operands. The AWG chip is designed with a frequency spacing of 100 GHz, allowing for a theoretical maximum modulate rate of the input vector of 50 GHz, which can be achieved through high-speed ring resonator modulators. The total footprint of the entire AWG structure is 1.5 mm × 1.5 mm. The area occupied by the number of modulation devices (*M* + *N*) can be disregarded compared to the quadratic growth of the number of operations.

Indeed, despite the traditional perception of AWGs being known for their large footprint, we have successfully harnessed their unique characteristics to overcome the inherent redundancy in convolution operations. This has led to outstanding compute density performance metrics in the photonic convolutional computing architecture.

**Figure 6:**
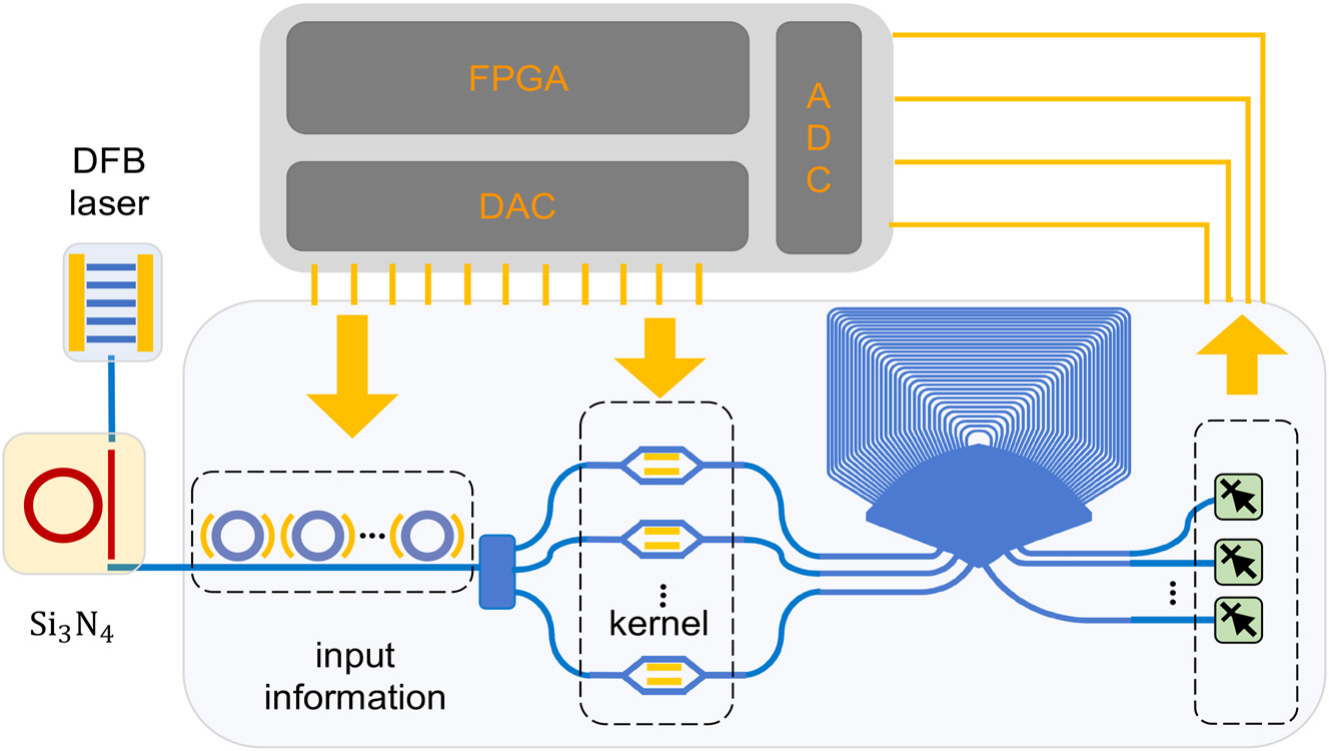
AWG photonic convolver based on the monolithic platform. FPGA, field programmable gate array; DAC, digital-to-analog converter; ADC, analog-to-digital converter.

We currently employ a spectral shaper for low-speed processing of multi-wavelength signals due to the constraints of high-speed interfaces. However, with the advancements in commercial silicon photonics processing platforms, the future holds potential for more integrated on-chip implementations. Figure 6 presents a perspective of such an AWG photonic convolver in the future. This implementation is based on the same architecture as our current experiment, but it utilizes a monolithic platform to increase computation speed and energy efficiency by potential orders of magnitude. In this proposed setup, a multi-wavelength optical frequency comb is generated using a distributed feedback laser combined with a silicon nitride micro-cavity. This multi-wavelength signal is then introduced into the silicon photonics chip. It first traverses a swift carrier depletion micro-ring array for input vector encoding. Following beam splitting, the signal undergoes low-speed convolutional kernel encoding facilitated by a thermally-tuned Mach-Zehnder interferometer. After the routing of AWG, it is finally captured by high-speed Si–Ge photodetectors. This system comprises a customized high-speed digital-to-analog converter chip, an analog-to-digital converter chip, and an FPGA chip, collaboratively working to achieve optimal optical convolution performance.

## 5 Conclusions

In summary, we demonstrate an integrated optical convolution architecture that leverages AWG to execute *M* × *N* multiply-accumulate (MAC) operations through *M* + *N* units, enabling the convolution to be completed directly within one clock cycle. This achievement marks for the first time a non-redundant convolution architecture on an integrated optical platform. In our experimental validation, we achieve a precision of 5 bits and obtain 91.9 % accuracy in a 10-class handwritten digit recognition task. The theoretical computing power density of our architecture reaches an outstanding 8.53 TOPS mm^−2^. Its ultra-low computational latency and cascading scalability not only significantly enhance the efficiency of optical convolution computation but also establish a solid foundation for future integrated all-optical convolutional neural networks, opening up promising avenues for more efficient and powerful optical computation platform.
